# Economic evaluation of smoking cessation in Ontario's regional cancer programs

**DOI:** 10.1002/cam4.1495

**Published:** 2018-07-17

**Authors:** Sandjar Djalalov, Lisa Masucci, Wanrudee Isaranuwatchai, William Evans, Alice Peter, Rebecca Truscott, Erin Cameron, Nicole Mittmann, Linda Rabeneck, Kelvin Chan, Jeffrey S. Hoch

**Affiliations:** ^1^ Health Quality Ontario Toronto Ontario Canada; ^2^ St. Michael's Hospital Toronto Ontario Canada; ^3^ Cancer Care Ontario Toronto Ontario Canada; ^4^ University of Toronto Toronto Ontario Canada; ^5^ Canadian Centre for Applied Research in Cancer Control Canada; ^6^ McMaster University Hamilton Ontario Canada; ^7^ Sunnybrook Health Sciences Centre Toronto Ontario Canada; ^8^ University of California, Davis Davis California

**Keywords:** Cost‐effectiveness, oncology, smoking cessation

## Abstract

Quitting smoking after a diagnosis of cancer results in greater response to treatment and decreased risk of disease recurrence and second primary cancers. The objective of this study was to evaluate the potential cost‐effectiveness of two smoking cessation approaches: the current basic smoking cessation program consisting of screening for tobacco use, advice, and referral; and a best practice smoking cessation program that includes the current basic program with the addition of pharmacological therapy, counseling, and follow‐up. A Markov model was constructed that followed 65‐year‐old smokers with cancer over a lifetime horizon. Transition probabilities and mortality estimates were obtained from the published literature. Costs were obtained from standard costing sources in Ontario and reports. Probabilistic and deterministic sensitivity analyses were conducted to address parameter uncertainties. For smokers with cancer, the best practice smoking cessation program was more effective and more costly than the basic smoking cessation program. The incremental cost‐effectiveness ratio of the best practice smoking cessation program compared to the basic smoking cessation program was $3367 per QALY gained and $5050 per LY gained for males, and $2050 per QALY gained and $4100 per LY gained for females. Results were most sensitive to the hazard ratio of mortality for former and current smokers, the probability of quitting smoking through participation in the program and smoking‐attributable costs. The study results suggested that a best practice smoking cessation program could be a cost‐effective option. These findings can support and guide implementation of smoking cessation programs.

## Introduction

Smoking is a leading cause of mortality and the leading cause of preventable death in Canada 1. Smoking results in an increased risk of all‐cause and cancer‐specific mortality 2. At a global level, approximately nine million premature deaths per year may be attributed to smoking 3. In Ontario, Canada, approximately 77,000 new cancer cases are diagnosed each year 4. Approximately 20% of these persons are current smokers at the time of cancer diagnosis and 30–60% of them continue smoking after diagnosis 5. Evidence suggests that the risk of dying from cancer could be lowered by 30–40% by quitting smoking at the time of cancer diagnosis 2. Quitting smoking at diagnosis improves prognosis and results in improved general health, reduced toxicity from treatment, greater response to treatment and decreased risk of disease recurrence and second primary cancers 6.

Illnesses that result from smoking are responsible for considerable health care costs, as well as loss of productivity from work. The societal cost of tobacco use in Canada in 2002 was estimated to be approximately 17 billion Canadian dollars 7. According to a report published by the Institute for Clinical Evaluative Sciences and Public Health Ontario, smoking results in a loss of an average of 2.0–2.5 years of life expectancy for Ontarians 8. As a result of the considerable social and economic burden of smoking, governments have undertaken smoking cessation initiatives with the objective of lowering the smoking prevalence rates 7. Initiatives have included media campaigns on the adverse effects of smoking, tax increases and packaging/marketing of tobacco products, telephone quit‐lines for cessation support and school and community‐based programs 9.

Pharmacological interventions and counseling are important components of a smoking cessation program and evaluations of these programs have consistently demonstrated the effectiveness and importance of physician based‐interventions. Cancer Care Ontario, the provincial agency responsible for improving cancer services in Ontario, is currently implementing a best practice smoking cessation intervention for new ambulatory cancer patients presenting to its 14 Regional Cancer Programs. All 14 Regional Cancer Programs are currently screening patients for smoking status, advising on the benefits of quitting and offering referrals to smoking cessation resources. Smokers are advised that quitting tobacco use will benefit their general health, improve cancer treatment outcomes, and reduce the chance of developing comorbidities. Data on screening and other performance metrics have been collected but do not include information on physician follow‐up rates, quit rates, or the use of pharmacological interventions 10.

Value for money is an important consideration in public policy decisions and influences decision‐makers in how to allocate resources across the health care system. An economic evaluation can help inform the costs and effects of implementing a smoking cessation initiative within Regional Cancer Programs. Much has been published on the cost‐effectiveness of smoking cessation at the population level but there is almost no information related to the oncology setting. A recent systematic literature search found only one study examining the cost‐effectiveness of a smoking cessation program implemented at the time of surgery for lung cancer 11. The lack of economic evidence to inform decision‐makers on the cost‐effectiveness of smoking cessation programs in persons with a new diagnosis of cancer motivated this study.

The objective of this study was to evaluate the potential cost‐effectiveness of two smoking cessation programs: the current basic smoking cessation program and a best practice smoking cessation program to guide implementation of smoking cessation programs across Ontario's cancer system.

## Methods

### Treatment strategies and target population

We compared two smoking cessation programs: the current basic approach to smoking cessation in Regional Cancer Programs, which includes only screening, advice and referral; and a best practice approach, which includes the basic program plus pharmacological therapy (specifically varenicline), counseling (once a week for 15 min with a smoking cessation nurse over 12 weeks), and follow‐up (Fig. S1) 12. Our target population was Ontario cancer patients aged 65 years. Individuals, 65 years were selected as the cohort of interest as that is the average age of cancer patients within the Cancer Care Ontario smoking cessation program.

### Model

**Figure 1:**
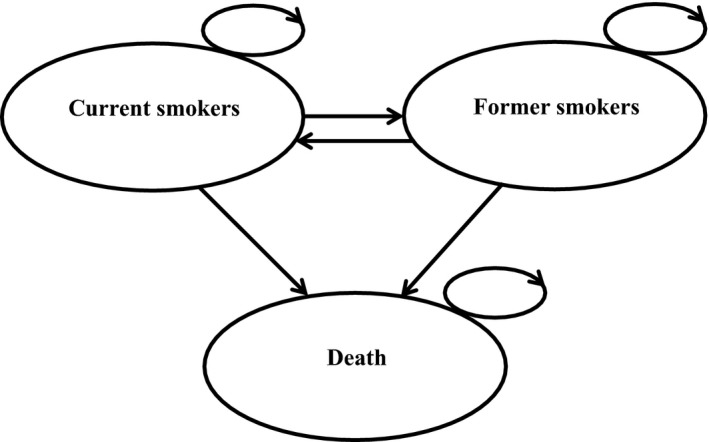
Decision analytic model.

A Markov model, with yearly cycle lengths, was developed to simulate lifetime health profiles and to compare the two cessation programs in a cohort of 65‐year‐old current smokers with cancer (Fig. 1). There were three main states in the model: current smokers, former smokers, and death. The direction of the arrows indicates possible transitions between these three states. Cancer patients who were successful with smoking cessation became former smokers and from this state, they could either remain a former smoker, relapse to become a smoker again, die from tobacco‐related disease or die from a non‐tobacco‐related cause. Smokers who were unsuccessful in their quit attempt remained in the current smoker state. The model assumes that current smokers could undertake a self‐quit initiative; therefore, there was a potential for transition from the smoker state to the former smoker state in the absence of a smoking cessation program. We used data from the literature (Table 1) to assign costs, transition probabilities, and quality of life utility estimates to each health state over a lifetime time horizon. Health benefits were expressed as quality‐adjusted life‐years gained and life‐years gained. The incremental cost‐effectiveness ratio (ICER) was calculated by dividing the difference in expected costs by the difference in expected health outcomes between the two programs. Both costs and outcomes were discounted at 5% per annum 13. The cost‐effectiveness analysis was conducted from the perspective of a single healthcare payer.

**Table 1:** Variables used in the model: probabilities, costs, and utilities

Variable	Base case	Lower range	Upper range	Source
12‐month abstinence rate in best practice program	0.24	0.14	0.36	Ong et al. 2016 14
12‐month abstinence rate in basic program	0.04	0.03	0.05	Fiscella and Franks 1996 15, and Orme et al. 2001 16
Quit due to smoking cessation program in year 2	0.02	0.02	0.03	Taylor et al. 2014 and Coleman et al. 2010
Self‐quit	0.015	0.012	0.018	Stapleton et al. 1999 20
Long‐term relapse rates for former smokers				
1–2 years	0.24	0.19	0.29	Gilpin, Pierce, and Farkas 1997 32, Yudkin et al. 2003 33 and Wetter et al. 2004 34
3–4 years	0.10	0.08	0.12	
5–8 years	0.02	0.016	0.024	
9–10 years	0.021	0.017	0.025	
10+ years	0.005	0.004	0.006	
Mortality hazard ratio				
Current cancer smokers versus never	1.50	1.07	1.50	Lee et al. 2014 21
Former cancer smokers versus never	1.30	0.95	1.81	
Background mortality	Canadian life tables			Statistics Canada
Cancer‐related mortality	Canadian vital statistics			Canadian vital statistics death database
Smoking cessation nurse fee	$105	$95	$116	Average registered nurse salary in Ontario $35/h (15 min × 12 session) 24
Best practice program administration cost (per patient; one‐time cost)	$47	$33	$61	CCO SCP budget proposal 10
Basic program administration cost (per patient; one‐time cost)	$16	$15	$18	CCO SCP budget proposal 10
Pharmacological therapy (Varenicline) (one‐time cost)	$150	$105	$195	Ontario drug benefit 35
Annual cancer patient health care cost after diagnosis	$25,058	$24,897	$25,219	de Oliveira et al. 2013 26
Smoking‐attributable annual cost	$403	$262	$486	Norouzi 2012 25
Time horizon	Lifetime	2	4	Authors’ assumption
Utilities				
Former smoker: women				
65–74	0.7709	N/a	N/a	Vogl et al. 2012 22
75–100	0.6981			
Current smoker: women				
65–74	0.7496	N/a	N/a	Vogl et al. 2012 22
75–100	0.6753			
Former smoker: men				
65–74	0.7802	N/a	N/a	Vogl et al. 2012 22
75–100	0.7358			
Current smoker: men				
65–74	0.7551	N/a	N/a	Vogl et al. 2012 22
75–100	0.7089			
Utility decrement due to cancer	0.12	0.11	0.13	Mittmann et al. 1999 23

### Transition probabilities

The 12‐month abstinence rate as a result of the best practice smoking cessation program was obtained from a study examining the impact of motivational interviewing, regular follow‐up and pharmacotherapy among patients diagnosed with potentially curable cancer 14. After 12 months, patients experienced a lower quit rate. The 12‐month abstinence rate for the basic smoking cessation program was obtained from three studies which consisted of physician advice and pharmacotherapy 15, 16, 17. After 12 months, we took into a consideration a self‐quit rate for the basic smoking cessation program. The background annual probability to quit and the long‐term relapse rates were obtained from the clinical literature 18, 19, 20.

We assumed that all‐cause mortality in cancer patients included cancer and non‐cancer related death. As the probability of a cancer death differs between males and females, we separated the analysis by sex. As there was no direct source of Canadian cause‐specific mortality stratified by smoking status, we took the following steps to calculate the mortality rate by smoking status for cancer patients: Step 1, identified the age‐specific probability of death for male and females in Ontario; Step 2, identified the age‐specific percentage of cancer and non‐cancer deaths from all‐cause mortality life tables; Step 3, calculated the probability of death for cancer and non‐cancer status using non‐cancer death for all‐cause mortality; Step 4, identified the mortality by age, smoking status, and years of abstinence from literature; Step 5, calculated the relative risk of mortality by age and smoking status; and Step 6, derived the adjusted mortality rates by age, sex, and smoking status. Data for the Ontario population were obtained from Statistics Canada and the Canadian Vital Statistics Death database. We obtained the hazard ratio of mortality at 12 months related to tobacco use among patients with lung cancer and by smoking status 21.

### Utilities

We incorporated health‐related quality of life into the model using utilities as a measure of the health state value for each year by smoking status, age, and sex. Health state values were obtained from a study that measured the EQ‐5D among smokers, former smokers, and never smokers in a general population in the United Kingdom (*n* = 13,241) 22. We applied a 0.12 utility decrement for the cancer patients. This was obtained from a study examining the health utilities of 20 chronic conditions in the Canadian population (*n* = 17,626) reported in the National Population Health Survey 23.

### Costs

We estimated costs from standard costing sources in Ontario, Canada, and the published literature. The cost of counseling was based on a total of 12 sessions (15 min per session over 12 weeks) with a smoking cessation nurse 24. Program administration costs were based on a budget proposal developed by Cancer Care Ontario for a smoking cessation program in the Regional Cancer Programs 10. The program included the establishment of a Community of Practice, provision of evidence‐based resources for program leads, funding for one full‐time tobacco cessation counselor to provide onsite coaching, and evaluation of the program in each of the 14 regions of the province. The program administration cost per cancer patient who smoked was calculated by dividing the budgeted annual cost by the estimated number of new cancer patients smoking in Ontario in 2014. We used a conservative approach to determine the cost of cessation medications by choosing one of the more expensive pharmacological treatments; namely, varenicline (1 mg for 12 weeks), which is covered by the Ontario Drug Benefit for individuals 65 years of age 25. We assumed that all patients in the best practice smoking cessation program took the drug as prescribed and attended all follow‐up visits. The annual cancer patient health care costs after diagnosis were based on a population‐based descriptive study of the 21 most common cancers in Ontario 26. An estimation of the smoking‐attributable hospital and physician healthcare resource utilization rates were based on a study that obtained the health service utilization of smokers from the 2012 Canadian Community Health Survey. Hospitalization, physician, and nurse costs were then applied to these health care utilization rates. Costs were reported in 2015 Canadian dollars. To inflate costs to 2015, the Consumer Price Index for health care services in Ontario was used 27.

### Sensitivity analysis

We conducted deterministic and probabilistic sensitivity analyses to assess the uncertainty of all parameters. We conducted a one‐way sensitivity analysis on key model parameters such as time horizon, abstinence rates, quit rates, relapse rates, relative risk of mortality, utility decrement, and costs (ranges shown in Table 1). For the probabilistic sensitivity analyses, we used gamma distributions to represent uncertainty in the cost parameters because cost data are typically skewed and cannot be negative. We used beta distributions for the probabilities and utilities because these estimates were confined to a 0–1 range. Lognormal distributions were used for the hazard ratio of mortality for current and former smokers (Table 1). All parameters were randomly sampled from their assigned distributions, and 10,000 simulations were performed. We estimated the likelihood of each treatment strategy being more favorable across a range of Willingness‐to‐Pay thresholds using cost‐effectiveness acceptability curves (CEAC). We also summarized the results on a cost‐effectiveness plane.

## Results

### Base case results

In cancer patients, the best practice smoking cessation program for smokers was more effective (0.03 QALYs gained for males and 0.02 for females) and more costly (an additional $101 per patient for males and $41 per patient for females) than the basic smoking cessation program. For males, the incremental cost‐effectiveness ratios of the best practice smoking cessation program was $3367 per QALY gained and $5050 per LY gained and for females, $2050 per QALY gained and $4100 per LY gained (Table 2).

**Table 2:** Incremental cost‐effectiveness of smoking cessation in the regional cancer programs of Ontario

Strategy	Male			Female		
	Cost ($)	QALY (discounted)	LY (discounted)	Cost ($)	QALY (discounted)	LY (discounted)
Basic smoking cessation program	294,859	7.30	11.60	325,638	7.83	12.80
Best practice smoking cessation program	294,960	7.33	11.62	325,679	7.85	12.81
Incremental	$101	0.03	0.02	$41	0.02	0.01
ICER		$3367/QALY	$5050/LY		$2050/QALY	$4100/LY

ICER, Incremental cost‐effectiveness ratio; LY, life‐year; Program; QALY, quality‐adjusted life‐year.

### Deterministic sensitivity analysis

**Figure 2:**
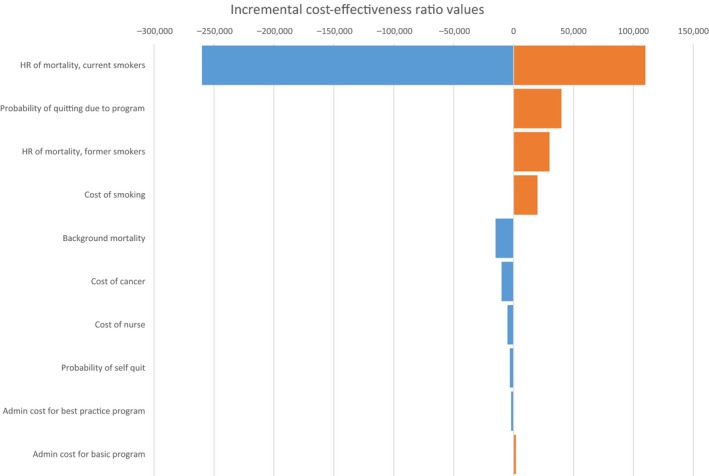
One‐way sensitivity analysis ICER, Incremental cost‐effectiveness ratio.

One‐way sensitivity analysis revealed that the results were most sensitive to the hazard ratio for mortality for former and current smoker cancer patients, the annual health care cost of cancer patients after diagnosis, the probability of quitting due to the basic smoking cessation program, and smoking‐attributable cost (Fig. 2). The impact of changes in other parameters, such as program administration cost and the probability of self‐quit were less pronounced. When we shortened the time horizon to 2 years, the best practice smoking cessation program remained more costly and more effective. We also explored a scenario that excluded the annual health care costs of cancer patients in both arms of the model. In this scenario, the best practice smoking cessation program again remained more costly and more effective.

### Probabilistic sensitivity analysis

**Figure 3:**
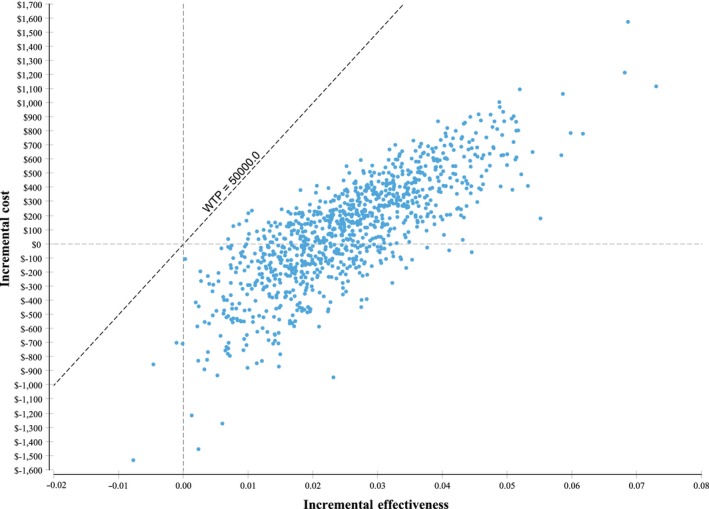
Probabilistic sensitivity analysis results on the cost‐effectiveness plane. QALYs, Quality‐adjusted life years.

**Figure 4:**
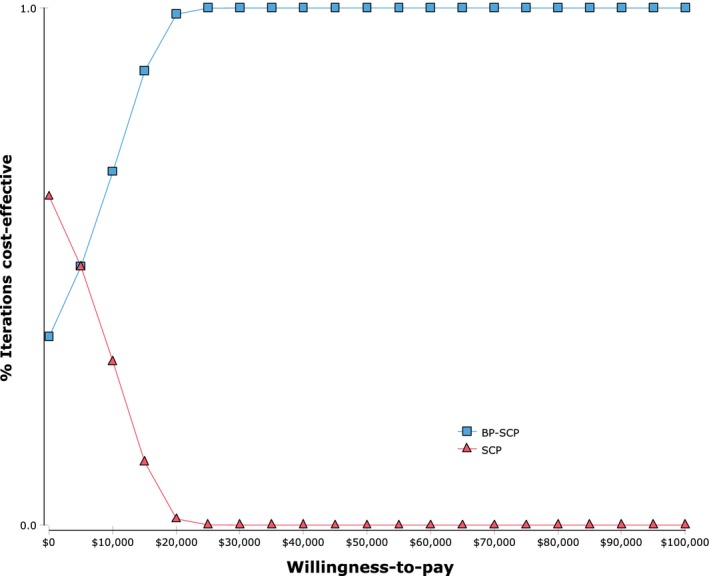
Cost‐effectiveness acceptability curve. BP‐SCP, Best practice‐smoking cessation program; SCP, Basic‐smoking cessation program.

Probabilistic sensitivity analysis revealed that the 10,000 simulated ICERs were located in either the northeast quadrant meaning that the intervention was both more costly and more effective or the southeast quadrant meaning that the intervention was less costly and more effective (Fig. 3). If one QALY gained was valued at Can $50,000, then 100% of the simulated ICERs were considered cost‐effective (Fig. 4).

## Discussion

Our results are consistent with previous economic evaluations that used a similar model structure and intervention 28, 29. These models showed that among the range of interventions, counseling (with follow‐up) plus pharmacological therapy was the most cost‐effective intervention compared to nicotine patch 30, 31. A smoking cessation program implemented at the time of surgery for lung cancer has been shown to be a highly cost‐effective intervention in a US setting 11.

This study should be interpreted in light of its strengths and limitations. First, this study was exploratory in nature as data were based on a smoking cessation pilot project with limited data on physician follow‐up and quit rates. We selected the studies that were most applicable to the smoking cessation programs offered at the Ontario regional cancer center. As such, we relied on clinical evidence from broad populations in other countries. We believe these studies closely reflect the services offered at the cancer centers. This study may not be generalizable to other regions that have a different program. Second, we used the hazard ratio of current and former smokers’ mortality compared to never smokers to show the benefit of the smoking cessation program. This parameter was quite sensitive and, therefore, splitting the risk of death between cancer attributable and non‐cancer death could have had a major effect on our results. Third, the model did not take into consideration the outcomes and cost of other diseases and their comorbidities, such as stroke, chronic obstructive pulmonary disease, and cardiovascular diseases associated with smoking. If these comorbidities were taken into consideration, the best practice smoking cessation program could be even more economically attractive, as smokers are more likely to have other smoking‐related comorbidities and higher health care costs, which we have not included in our model. As well, our model considers the cancer patient in “general,” without specifying the particular cancer type or severity. Healthcare resource utilization and mortality vary widely depending on the type of cancer and severity, which could affect the cost‐effectiveness of the smoking cessation intervention. Lastly, the model did not take into account any of the potential benefits from the reduction in “second‐hand smoke” exposure to others 28. Although it is difficult to measure the risk from environmental tobacco exposure, abstinence from smoking would definitely improve conditions for family members and potentially others.

Further research on smoking‐attributable mortality in cancer patients is required to improve the validity of the parameters used in the model. This is an exploratory analysis with many assumptions. It would be important to conduct an economic evaluation based on observed data from a specific Regional Cancer Program site in order to better understand the value for money of a best practice smoking cessation intervention.

In conclusion, a best practice smoking cessation program for cancer patients has the potential to be an economically attractive option when compared to a basic smoking cessation program over a broad range of assumptions. From this analysis, it appears that public funding for a best practice smoking cessation program for cancer patients in Ontario might be a promising way to reduce economic and healthcare burden from smoking in the ambulatory oncology setting.

## Conflict of Interest

There is no conflict of interests to declare.

## Supporting information


**Figure S1**. Smoking cessation programs.
