# Supplementary figures and images for: Economic evaluation of smoking cessation in Ontario's regional cancer programs
Source: Cancer Med. 2018 Jul 17;7(9):4765–72. doi: 10.1002/cam4.1495 (PMC6144163; doi:10.1002/cam4.1495)

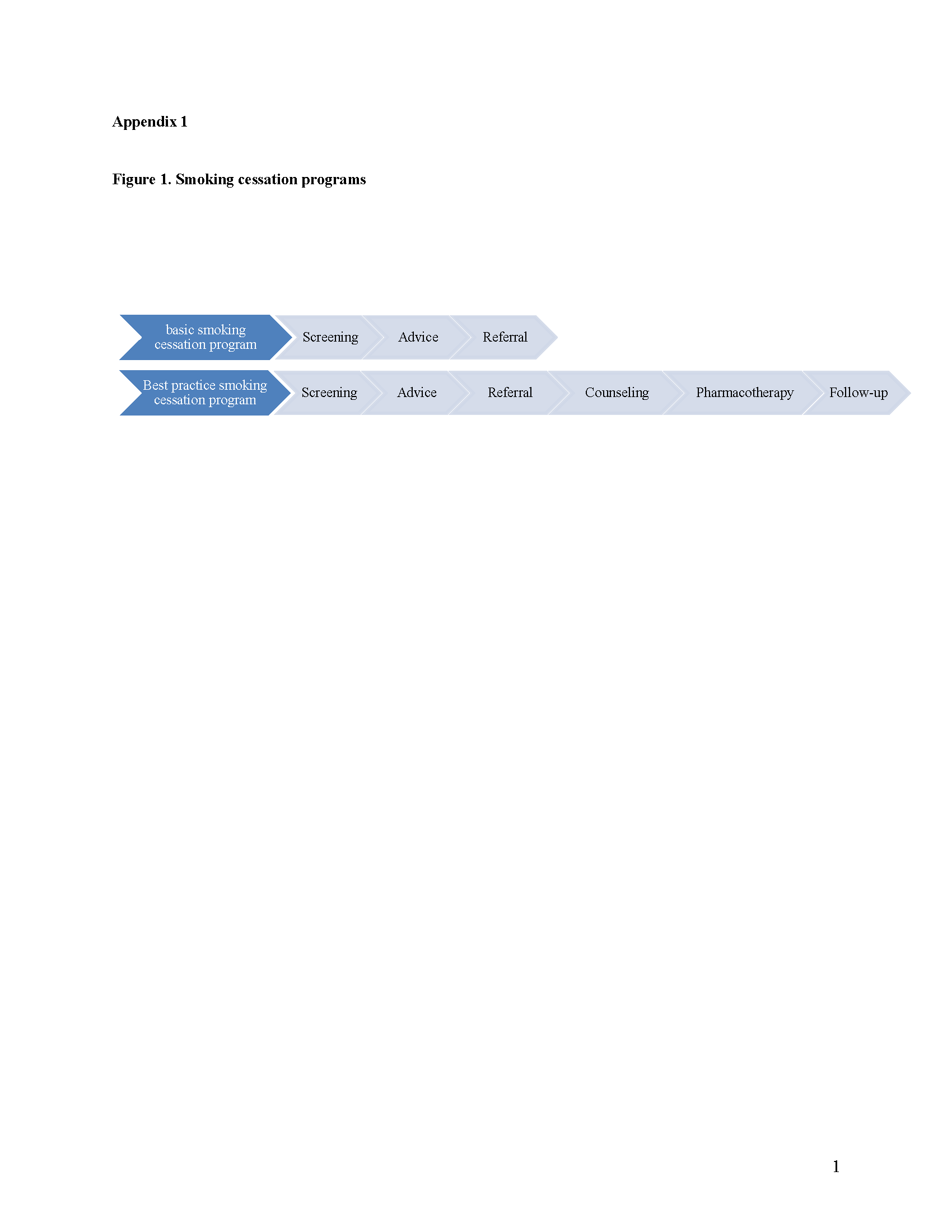

Supplement: Supplementary file 1 — Figure S1. Smoking cessation programs. [file CAM4-7-4765-s001.tif]
